# Draft Genome Sequence of *Bacillus* sp. Strain CMAA 1185, a Cellullolytic Bacterium Isolated from Stain House Lake, Antarctic Peninsula

**DOI:** 10.1128/genomeA.00436-15

**Published:** 2015-05-28

**Authors:** Suikinai Nobre Santos, Vanessa Nessner Kavamura, Rodrigo Gouvêa Taketani, Rafael L. F. Vasconcellos, Tiago Domingues Zucchi, Itamar Soares Melo

**Affiliations:** a Laboratory of Environmental Microbiology, Embrapa Environment, Brazilian Agricultural Research Corporation, EMBRAPA, Jaguariúna, São Paulo, Brazil; b Agrivalle, Pouso Alegre, Minas Gerais, Brazil

## Abstract

The aim of this study was to report the genome sequence of the cellulolytic *Bacillus* sp. strain CMAA 1185, isolated from Stain House Lake, Antarctica.

## GENOME ANNOUNCEMENT

Antarctica has become an interesting site for bioprospecting microorganisms with biotechnological purposes. This extremely harsh environment usually poses numerous threats to living beings (1) and, notwithstanding, may select organisms that held unique biosynthetic pathways (2). In our survey at Stain House Lake, we isolated bacteria able to degrade cellulose (3). One of these bacterial isolates, CMAA 1185, was revealed to produce a cellulolase active at low temperatures, which may play a crucial, active at low temperatures, which may play a crucial role in this environment, and it also has many industrial applications. Thus, to extend the knowledge of the genes related to hydrolases of biopolymers of strain CMAA 1185, whole-genome sequencing was performed using the Ion Torrent (PGM) platform. Genomic DNA was extracted from a pure culture grown overnight on LB medium using the PureLink genomic DNA kit (Life Technologies). Sequencing was carried out on the Ion 316 Chip sequencer provided in the Ion sequencing kit 200-bp version 2.0, according to the manufacturer’s protocol. The genome sequence was *de novo* assembled using the MIRA version 4, CLC Genomics Workbench version 5.5.1, and SeqMan NGen version 4.0.0 packages, and the contigs obtained from the assembly were integrated using CISA (4, 5). The taxonomic position of strain CMAA 1185 was further evaluated using the JSpecies package (6).

The number of reads (<Q20) generated using *Bacillus subtilis* subsp. Natto BEST195 (GenBank accession no. PRJDB2126) as a reference was 2,816,934, which were allocated into 10 contigs, with 136× coverage and a mean length of 1,046,675 bp. The assembled data were analyzed by RAST annotation (7), and the genome size was found to be 3,907,872 bp, comprising 4,263 open reading frames (ORFs). The G+C content was estimated to be 43.3 mol%.

A total of 4,051 genes were identified. Of these, there are 176 pseudogenes, 12 rRNAs genes (5S, 16S, and 23S), 65 tRNA genes, and one noncoding RNA (ncRNA) gene. The genome contains 17 genes copies related to hydrolyzes, mainly glycosyltransferases, polysaccharide lyase, peptidoglycan chitinase A, and amylase (8). 16S rRNA analyses revealed that this isolate is most closely related to the type strain of *Bacillus subtilis* (3). These organisms share an average nucleotide identity of 99%.

### Nucleotide sequence accession numbers. 

This whole-genome shotgun project has been deposited at DDBJ/EMBL/GenBank under the accession no. LAKC00000000. The version described in this paper is version LAKC01000000.
